# Ovarian cancer detection from metabolomic liquid chromatography/mass spectrometry data by support vector machines

**DOI:** 10.1186/1471-2105-10-259

**Published:** 2009-08-22

**Authors:** Wei Guan, Manshui Zhou, Christina Y Hampton, Benedict B Benigno, L DeEtte Walker, Alexander Gray, John F McDonald, Facundo M Fernández

**Affiliations:** 1 College of Computing, Georgia Institute of Technology, Atlanta GA 30332, USA; 2 School of Chemistry and Biochemistry, Georgia Institute of Technology, Atlanta GA 30332, USA; 3 Ovarian Cancer Institute, Atlanta, GA 30332, USA; 4 School of Biology, Georgia Institute of Technology, Atlanta GA 30332, USA

## Abstract

**Background:**

The majority of ovarian cancer biomarker discovery efforts focus on the identification of proteins that can improve the predictive power of presently available diagnostic tests. We here show that metabolomics, the study of metabolic changes in biological systems, can also provide characteristic small molecule fingerprints related to this disease.

**Results:**

In this work, new approaches to automatic classification of metabolomic data produced from sera of ovarian cancer patients and benign controls are investigated. The performance of support vector machines (SVM) for the classification of liquid chromatography/time-of-flight mass spectrometry (LC/TOF MS) metabolomic data focusing on recognizing combinations or "panels" of potential metabolic diagnostic biomarkers was evaluated. Utilizing LC/TOF MS, sera from 37 ovarian cancer patients and 35 benign controls were studied. Optimum panels of spectral features observed in positive or/and negative ion mode electrospray (ESI) MS with the ability to distinguish between control and ovarian cancer samples were selected using state-of-the-art feature selection methods such as recursive feature elimination and L1-norm SVM.

**Conclusion:**

Three evaluation processes (leave-one-out-cross-validation, 12-fold-cross-validation, 52-20-split-validation) were used to examine the SVM models based on the selected panels in terms of their ability for differentiating control vs. disease serum samples. The statistical significance for these feature selection results were comprehensively investigated. Classification of the serum sample test set was over 90% accurate indicating promise that the above approach may lead to the development of an accurate and reliable metabolomic-based approach for detecting ovarian cancer.

## Background

Despite decades of research and an annual investment in the U.S. of more than $2 billion on treatment, ovarian cancer remains the leading cause of deaths from gynecological malignancies [1]. It is estimated that 21,650 new cases of ovarian cancer were diagnosed in 2008 and 15,520 women died from the disease [2]. Due to the asymptomatic nature of the disease, women are frequently undiagnosed until the disease is late in its progression (stage III/IV) when the 5-year survival rate is only 15–20% [3]. The assay for CA125 is currently the only FDA-approved test for ovarian cancer detection but the overall predictive value of CA125 has been reported to be less than 10% [4].

Although screening for specific biomarkers that are diagnostic of ovarian cancer has been an active area of research since the early 1970's [5], no effective diagnostic tests are yet available. Most ovarian cancer biomarker discovery studies are based on the univariate or multivariate comparison of high throughput data focusing on qualitative or quantitative changes (e.g. methylation, glycosylation) of large biopolymers (e.g. DNA, RNA, glycans and proteins) [6]. In contrast, metabolic biomarker discovery approaches that focus on small molecules (below 1 kDa) have received significantly less attention, despite the fact that metabolic profiling of human serum has long been touted as a promising technology for the early detection of many diseases, including cancer [3]. In this trend, a few studies have reported individual metabolites potentially useful for ovarian cancer detection, the most studied being lysophosphatidic acid [7-9] and lipid associated sialic acid [10-14].

Since metabolites have vastly-differing chemical properties and occur in a wide range of concentrations, mass spectrometry (MS) is a preferred method for broadband metabolic profiling [15]. Although MS has been successfully applied in the development of proteomic biomarker panels using surface-enhanced laser/desorption ionization (SELDI) MS [4,16-18] and matrix-assisted laser desorption/ionization (MALDI) MS [19,20], technologies such as LC MS for the effective analysis of the metabolome are still evolving [21] as are bioinformatic techniques for the analysis of the resulting data [22].

In machine learning, SVMs [23] are widely considered to represent the state of the art in classification accuracy. Recently, SVMs have been applied to the supervised classification of cancer versus control sample sets from data obtained using SELDI MS [24-27], MALDI MS [28,29], gas chromatography (GC) MS [30], LC/Quadrupole Linear Ion Trap MS [31], and LC/Ion Trap MS [32]. Other methods that have been used in supervised classification in chemometrics for cancer detection include partial least squares-discriminant analysis (PLSDA) [33,34], soft independent modeling of class analogy (SIMCA) [35], artificial neural networks (ANNs) [36], and classification and regression trees (CART) [37]. During classification, it is beneficial to perform feature selection (reduce the number of predictor variables) in order to make the diagnostic process cheaper and targeted, and to narrow down the number of biomarkers to better understand their biological significance. Feature selection allows the identification of robust spectral features that may otherwise be obscured by biological variability not related to disease. It has been shown that reducing the number of variables used for supervised multivariate model building is also beneficial for eliminating non-informative data, reducing prediction errors, and simplifying the interpretability of the data analysis results. For example, SVMs have been successfully combined with Information Gain and ReliefF [31] and Oscillating Search Algorithm for feature selection [32] to select out metabolic markers in prostate cancer, and to improve prediction performance of breast cancer datasets, respectively.

In this paper we present, to the best of our knowledge, the first application of SVMs and SVM-related feature selection methods (recursive feature elimination (RFE) with linear and nonlinear kernel [38], L1SVM [39], and Weston's method [40]) for classifying LC/TOF MS data of serum samples from ovarian cancer patients and controls. The statistical confidence of the prediction performance results was further assessed through hypothesis testing, and the general performance of the feature selection methods was extensively tested. The results demonstrate the utility of this approach to derive panels of metabolic spectral features that are potentially useful for the diagnosis of ovarian cancer.

## Methods

### Cohort Description

Serum samples were obtained from 37 patients with papillary serous ovarian cancer (mean age 60 years, range 43–79, stages I–IV) and 35 controls (mean age 54 years, range 32–84). The control population consisted of patients with histology considered within normal limits (WNL) and women with non-cancerous ovarian conditions. The patients' information is detailed in Table 1. All serum samples were obtained from the Ovarian Cancer Institute (OCI, Atlanta, GA) after approval by the Institutional Review Board (IRB). All donors were required to fast and to avoid medicine and alcohol for 12 hours prior to sampling, except for certain allowable medications, for instance, diabetics were allowed insulin. Following informed consent by donors, 5 *mL *of whole blood were collected at Northside Hospital (Atlanta, GA) by venipuncture from each donor into evacuated blood collection tubes that contained no anticoagulant. Serum was obtained by centrifugation at 5000 rpm for 5 minutes at 4°*C*. Immediately after centrifugation, 250 *μL *aliquots of serum were frozen and stored at -80°*C *for further use. The sample collection and storage procedures for both ovarian cancer patients and control individuals were identical.

**Table 1:** Characteristics of Ovarian Cancer Patients and Controls

**Characteristics**	**Stages I/II**	**Stages III/IV**	**Controls**	**Total**
Age (y), mean (range)	60(43–74)	61(46–79)	54(32–84)	58(32–84)

Papillary Serous Carcinoma	9	28	0	37

Control	0	0	35	35

### Serum Sample Pretreatment and LC/TOF MS Analysis

A stock sample of human serum purchased from Sigma (S7023, St. Louis, MO) was used during the development of the serum sample pretreatment and LC/TOF MS analysis protocols. Upon arrival, the frozen sample was thawed and separated into 250 *μL *aliquots which were stored at -80°*C *for further use.

Serum samples were thawed, and proteins precipitated by addition of acetonitrile to the serum sample in a 5:1 ratio. The mixture was incubated at room temperature for 40 minutes and after centrifugation, the supernatant was retained and vacuum evaporated. The residue was reconstituted in 80% acetonitrile/0.1% TFA and 15 *μL *was injected onto a reverse phase analytical C18 column (Symmetry^®^, 3.5 *μm*, 2.1 × 150 *mm*, pore size 100Å, Waters, Milford, MA) installed in an Agilent 1100 Series LC system (Santa Clara, CA) coupled to a JEOL AccuTOF (Tokyo, Japan) mass spectrometer via an ESI source. Positive and negative ion mode ESI spectra were collected in the range of 100–1750 *m/z*. Every cancer sample was randomly paired with a control sample and run on the same day to ensure that no temporal bias was introduced. Sample pairs were run in random order and in duplicate. To ensure maximum reproducibility in metabolomic experiments, all serum specimens were run consecutively within a 2.5 month period. After LC/TOF MS analysis, the spectra were centroided, mass drift corrected, and exported in NetCDF format for further analysis. Method S-1 provides more detail about sample preparation and analysis, including the LC program used (Table S-1) [see Additional file 1].

### LC/TOF MS Data Preprocessing

All data were preprocessed identically and simultaneously. Preprocessing was performed by loading NetCDF files into mzMine (v0.60) [41]. Data were smoothed by chromatographic median filtering with a tolerance in *m/z *of 0.1, and one-sided scan window length of 3 *s*. Peaks were picked with a *m/z *bin size of 0.15, chromatographic threshold level of 0%, absolute noise level of 200, absolute minimum peak height of 250, minimum peak duration of 5 *s*, tolerance for *m/z *variation of 0.06, and tolerance for intensity variation of 50%. The method for de-isotoping was to assume +1 charge states, and monotonic isotopic patterns. The retention time tolerance (RT) for de-isotoping was 65 *s *and the *m/z *tolerance 0.07. The chromatographic peak alignment *m/z *tolerance was 0.2, and the RT tolerance was 12%, with a balance coefficient between *m/z *and RT of 30. The minimum number of detections for rare peak filtering in the alignment results was set to 41. Spectral features not initially detected by the peak detection algorithm were subsequently added by a gap filling method using an intensity tolerance of 30%, *m/z *tolerance size of 0.2, and RT tolerance size of 12%. Correction for systematic drift in intensity levels between different data files was performed by using linear intensity normalization of the total raw signal. After the normalized alignment file containing all peak intensities was created, peak areas were exported to Excel and peaks of contaminants, dimers, redundant adducts, and isotopes not adequately detected were removed. Approximately 37% of the peaks from positive mode and 18% of the peaks from negative mode were eliminated after this filtering step. Peak areas from duplicate runs were then averaged, and positive and negative mode ESI data were exported as ASCII files into Matlab for subsequent machine learning analysis. These data are available as a Matlab file, or as a set of text files [see Additional file 2].

### SVMs and Related Feature Selection Methods

SVMs [23] have been successfully applied to various scientific problems as they generally achieve classification performance superior to that of many older methods, particularly in high-dimensional settings [24-29]. Though computationally intensive, SVMs are efficient enough to handle problems of the size we consider here. Given a dataset  (*x*_*j *_∈ *R*^*N *^is the feature vector of *j*th instance and *y*_*j *_is the corresponding label), for a two-class classification problem, SVM finds the optimal separating hyperplane *w·x *+ *b *through the following quadratic optimization:

(1)

where function Φ(·): *R*^*n *^→ *U *maps the feature vector into high dimensional Euclidean subspace. Kernel function *K*(*x*_*i*_, *x*_*j*_) is defined as Φ(*x*_*i*_)·Φ(*x*_*j*_), for example, the linear kernel is *x*_*i*_·*x*_*j*_, a polynomial kernel is (*gx*_*i*_·*x*_*j *_+ *r*)^*d *^with parameters *g*, *r*, *d*. The above problem is usually solved through its dual formation [42].

Bagging strategies [43] are often used to boost the prediction performance of a classifier [44]. This approach involves generating multiple versions of a classifier and using these to obtain an aggregated predictor. A bagging process repeats the following procedure T times: i) bootstrap (sample from the dataset with replacement) from the training data to build a classifier and ii) obtain the predictions on the test data. The process then uses the majority voting results as the final predictions.

t2-statistics [45] is a widely used filter-based feature selection method in bioinformatics, calculated as  with degree of freedom , where *μ*_+_, *μ*_- _are the means and *δ*_+_, *δ*_- _are the standard deviations of the feature values, and *n*_+_, *n*_- _are the number of cancer patients and controls, respectively. Though computationally efficient, filter-based feature selection methods generally achieve inferior prediction performance compared to wrapper-based methods. Therefore, several SVM-based methods, such as the commonly used recursive feature elimination (RFE) method [38], were applied.

In RFE, the feature whose removal leads to a smaller increase to the cost function, *dJ*(*k*), is ranked as less important. , where *α *∈ *R*^*M *^is the dual variable vector of (1), *H*_*ij *_= *y*_*i*_*y*_*j*_*K*(*x*_*i*_, *x*_*j*_) and  with *x*^(-*k*) ^representing the feature vector with the *k*th feature removed. In the case of linear SVM, . At each RFE iteration, first, an SVM is trained with the currently selected feature set; next, the importance of the features is measured; then, the least important features are discarded successively from the remaining feature set. This procedure is repeated iteratively to study the prediction accuracy as a function of the number of remaining features and the smallest feature set that achieved the highest training accuracy is selected as the final output.

Bradley et al. [39] proposed L1SVM, which minimizes the L1-norm of the weight vector  rather than the L2-norm . Since the L1-norm is used, the optimal weight vector w is often very sparse, thus L1SVM can simultaneously perform classification as well as feature selection. However, this is only applicable in the case of the linear kernel. Previous literature suggest applying the standard L2-norm SVM on the feature selection results of L1SVM to improve the classification performance [46]. Fast algorithms for solving L1SVM were proposed by Fung & Mangasarian in 2004 [47] and Mangasarian in 2007 [48].

Weston et al. [40] introduced the idea of scaling variables, a feature is removed if the corresponding scaling variable *δ*_*k *_∈ {0, 1} becomes zero during the optimization. The scaling variables and the SVM are learned through minimizing a generalization error bound, *R*^2^*W*^2^, where *R*^2^(*β*, *δ*) is the radius of the smallest sphere, centered at the origin, that contains all the Φ(*x*_*i*_); *W*^2^(*α*, *δ*) is the L2 norm of the weight vector, and *δ *= (*δ*_1_,..., *δ*_*N*_)^*T *^is the vector of the scaling variables. The problem is approximated with an iterative method. At each iteration *t*, the algorithm firstly optimizes *R*^2^(*β*, *δ*^(*t*-1)^) and *W*^2^(*α*, *δ*^(*t*-1)^) separately (denoting the optimal solution as *α*^*t *^and *β*^*t*^, respectively); next, it minimizes *R*^2^(*β*^(*t*)^, *δ*)*W*^2^(*δ*^(*t*)^, *δ*) using gradient descent; then, it sets the smallest *δ*_*k *_to zero, i.e. removes the corresponding *k*th feature from the feature set. The above procedure repeats until only *d *nonzero scaling variable left.

### Statistical Significance Estimation

In addition to estimating the classification/feature selection performance using various cross-validation approaches, the statistical significance of these observations was further assessed through hypothesis testing. One possible non-parametric approach to hypothesis testing is permutation test, where no assumptions are made regarding the data distribution, and the p-value is computed as the cumulative sum using the empirical distribution. The permutation test works by comparing the statistic of interest with the distribution of the statistic obtained under the null (random) condition, and can be defined as follows [49]:

1. Repeat *T *times (where *t *is an index from 1, ⋯, *T*):

• Randomly permute the labels of the input data vectors.

• Compute the statistic of interest  for this permutation of labels, where  is the assigned label to *x*_*i *_at *t*^*th *^label randomization.

2. Compute the statistic of interest for the actual labels, *s*_0_.

3. Obtain the p-value : the cumulative probability of *s*_*t *_being greater than or equal to the observed statistics *s*_0_.

4. If the p-value <*α *(usually *α *= 0.05), reject the null hypothesis *H*_0_; otherwise, the observed result is not statistically significant.

### Metabolite Identification Procedure

Compound identification was attempted only for those spectral features remaining after the feature selection processes. Due to the biological complexity of serum samples, adduct ion analysis was first performed to ensure the unambiguous assignment of the signal of interest in each mass spectrum. Adducts formed in positive ion mode ESI usually include [*M *+ *H*]^+^, [*M *+ *NH*_4_]^+^, [*M *+ *Na*]^+^, [*M *+ *K*]^+^, [*M *- *H*_2_*O *+ *H*]^+ ^and [2*M *+ *H*]^+ ^species; in negative ion mode ESI [*M *- *H*]^-^, [*M *+ *CH*_3_*COO*]^-^, [*M *+ *Cl*]^-^, [*M *+ *HCOO*]^- ^and [2*M *- *H*]^- ^are generally observed. Adducts in centroided mass spectra corresponding to SVM-selected variables were identified by manually calculating the differences between the exact *m/z *values of peaks within the spectrum and comparing these differences to those between the common adduct species mentioned above. For spectra in which multiple adducts were not present, the accurate mass of the candidate neutral molecule was calculated based on the assumption that the peak of interest corresponded to either [*M *+ *H*]^+^, [*M *+ *Na*]^+^, or [*M *+ *NH*_4_]^+ ^in positive ion mode and [*M *- *H*]^-^, [*M *+ *CH*_3_*COO*]^-^, [*M *+ *HCOO*]^-^, or [*M *- *CH*_3_]^- ^(for glycerophosphocholines) in negative ion mode, yielding multiple candidate masses for each *m/z *value.

Elemental formulae were estimated from the accurate mass spectra using a freely distributed system of macros [50] that relies on a series of heuristic rules based on the mass accuracy of the peak of interest and the corresponding isotopic ratios. The mass of the neutral molecule and relative isotopic abundances were imported directly into the "seven golden rules" Excel spreadsheet [50]. The mass accuracy was set to 15 ppm, and the threshold for error in the relative isotopic abundances was set to 10%. The list of elements to include in the search was constrained to include C, H, N, O, P, S, Cl, and Br. The probability of a given formulae being the "correct" one is provided as a score calculated from the error rates in satisfying the aforementioned rules. The top hits in the list of filtered elemental formulae and all accurate mass values obtained were searched against the following databases: Metlin [51], KEGG [52], HMDB [53], MMCD [54] and Lipid Maps (LM) [55] in order to determine the greatest possible number of candidate molecules. The criteria used for the assignment of a tentative chemical structure were: a mass difference with the simulated formula lower than 15 ppm, isotope abundance errors less than 10%, and that the candidate found in the database corresponds to an endogenous metabolite.

## Results and discussion

### LC/TOF MS-based Metabolomic Analysis of Human Serum Samples

Metabolomic investigation of sera from patients with ovarian cancer and controls using LC/TOF MS revealed a total of 576 features extracted by mzMine in positive ion mode, and 280 in negative ion mode. The data were found to be highly complex, with numerous features across both analytical dimensions. Decreasing the absolute noise level and minimum peak height from 400 and 500 to 200 and 250 increased the number of detected features to 4439 and 329 for positive and negative ion modes, respectively. While this allowed us to "dig deeper" into the serum metabolome, the number of features consistently detected across samples decreased by 3.6% and 15%, respectively, suggesting that use of the previous settings provided a broad range of more stable features on which to base our feature selection methods. Detailed manual analysis of the entire dataset revealed the presence of additional redundant species (dimers, adducts, isotopes) that were removed, thus reducing the final number of features used to 360 positive ion mode and 232 negative ion mode features. We refer to the dataset with only positive ion mode features as "pos-ion-mode", the dataset with only negative ion mode features as "neg-ion-mode", and the dataset combining positive and negative ion mode features as "multimode", respectively.

**Figure 1:**
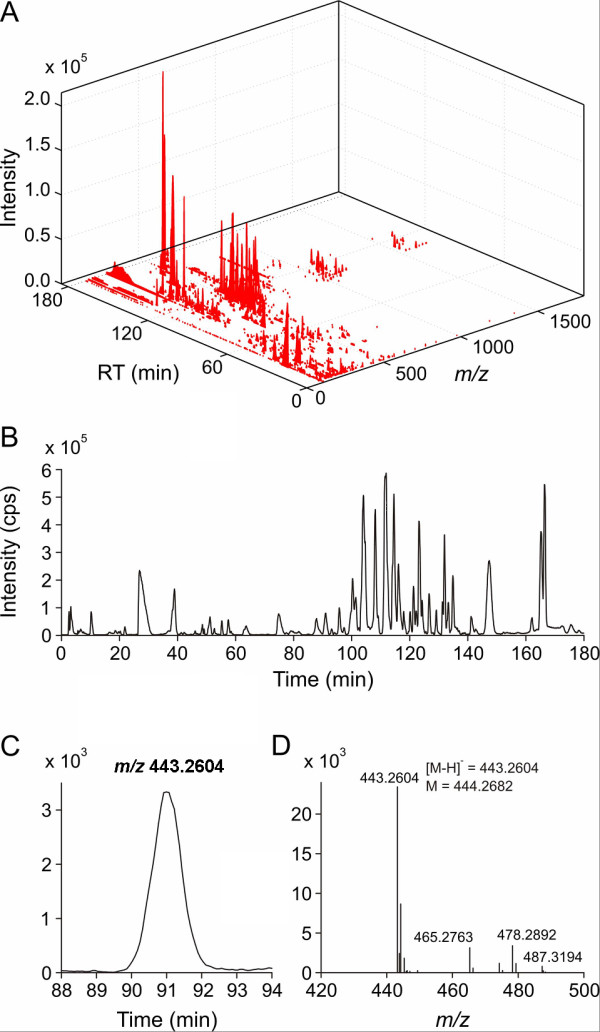
**LC/TOF MS-based Metabolic Analysis of Human Serum Samples**. Sample data obtained by negative ion mode LC/TOF MS analysis of a stage III ovarian cancer serum sample: (A) 3D intensity matrix, (B) total ion chromatogram, (C) selected i*m/z*on chromatogram for the feature at *m/z *443.26, and (D) mass spectrum at a retention time of 91 minutes.

A 3D serum metabolic profile for a typical stage III ovarian cancer serum sample is shown in Figure 1(a) demonstrating the capability of LC/TOF MS to resolve hundreds of compounds in a wide mass range within 180 minutes. Despite the shallow solvent gradient chosen for the LC run, there is still evidence of co-elution as observed in the projection of Figure 1(a) onto the chromatographic axis (Figure 1(b)). However, in most cases, the high resolving power of the TOF mass analyzer allowed the resolution of these signals by their selected ion chromatograms, as shown in Figure 1(c) for an ion with *m/z *= 443.26 at a window width of 0.05 *Da*. The corresponding centroided negative ion mode spectrum obtained at 91 minutes is shown in Figure 1(d). Due to the obvious complexity of these samples, the reproducibility of the LC/TOF MS approach was tested in early experiments to rule out column memory effects. Lipids, fatty acids and other hydrophobic components in sera that are easily adsorbed onto the reverse phase column can act as a new stationary phase, causing a change in selectivity, memory effects, and shifting retention times.

### Prediction Performance and Statistical Significance Analysis

SVMs and state-of-the-art feature selection methods were used to analyze the data. In the following sections, the linear SVM classifier is denoted as SVM, nonlinear SVM classifier with degree 2 polynomial kernel as SVM_NL; RFE feature selection with linear SVM as SVMRFE, RFE with nonlinear SVM as SVMRFE_NL, and Weston's feature selection method with nonlinear SVM as SVMRW. Three evaluation procedures were considered: i) leave-one-out-cross-validation (LOOCV); ii) 12-fold cross validation (12-fold CV) averaged over 10 trials (for each trial, the data were randomly ordered and split into 12 different folds and a 12-fold CV was performed); and iii) 52-20-split-validation averaged over 50 trials (for each trial, the data were randomly ordered and split into a training set of size 52 and a test set of size 20). All the three evaluation schemes were investigated for thoroughness, of these, LOOCV is expected to be the most reliable given the small sample size, therefore, we give the most detailed discussion regarding this scheme.

#### Prediction and Feature Selection Performance

The prediction performance for each dataset was first evaluated without feature selection. As apparent in Table 2, the nonlinear SVM classifier generally outperformed the linear SVM classifier, and the best prediction performance (83.3%) was obtained using the nonlinear SVM classifier in LOOCV evaluation. Although the neg-ion-mode dataset had a similar prediction performance as the multimode dataset, the analysis of sensitivity (how well cancer patients can be detected) and specificity (how well controls can be detected), somewhat favored usage of the latter, in that, the results showed that this dataset achieved a better balance between sensitivity and specificity (Tables 2). Therefore, only the results of multimode dataset are analyzed here, the results of the pos-ion-mode and neg-ion-mode datasets are shown in Table S-2 through S-5 [see Additional file 1].

**Table 2:** Prediction Performance (%) without Feature Selection (The last column lists the mean and standard deviation of the prediction performance (measured by the LOOCV average accuracy) over the permutation test (*T *= 1000))

Classifier	**52-20-split Validation** (50 trials)	**12-fold CV** (10 trials)	**LOOCV**			
			Accuracy	Sensitivity	Specificity	Permutation Test Results
SVM	75.8	80.3	81.9	81.8	81.6	49.5 ± 7.3

SVM_NL	76.3	81.7	83.3	86.5	80	49.5 ± 7.3

Next, the prediction performance was evaluated following feature selection. As discussed in the previous section, except for L1SVM, the other three feature selection methods are iterative methods with optimal feature sets determined according to criteria such as training accuracy (for SVMRFE, SVMRFE_NL), or generalization error bound (for SVMRW). In our experiments, a LOOCV average classification accuracy over the input dataset (for feature selection) containing only the selected feature subset was used as the criterion. The reasons are: i) the SVM training accuracy was almost always 100% until the feature set became unreasonably small and ii) the minimal generalization error was usually achieved when the feature set was quite large. The size of the feature set was further restricted to be less than 50 to allow for fair comparison of the performance with the L1SVM feature selection results.

**Figure 2:**
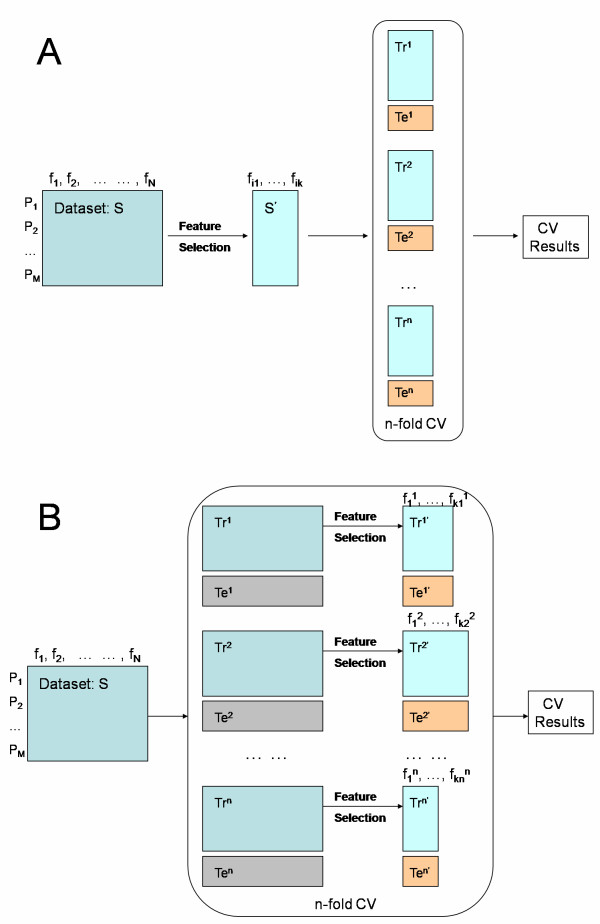
**Prediction Performance Evaluation Frameworks**. Evaluation frameworks for prediction performance subsequent to (A) feature selection applied to the whole dataset, and (B) feature selection applied to the training subsampling during each cross-validation.

In this second set of experiments (Figure 2(a)), each feature selection method was applied to the whole dataset, then the prediction performance of the dataset containing only the selected feature subset (panel) was measured using the three evaluation processes described above. The estimated predictive performance was surprisingly high (greater than 90%) under LOOCV (Tables 3 and 4), which is perhaps the most accurate evaluation technique in this low-sample setting. The feature selection results of SVMRFE_NL had the best discriminative power according to both LOOCV and 12-fold CV evaluation, while the feature subset selected by SVMRFE archived the best test accuracy in 52-20 split validation evaluation and the second best test accuracy in LOOCV and 12-fold CV evaluation.

**Table 3:** Prediction Performance (%): Feature Selection Methods Applied to the Whole Dataset (The last column lists the mean and standard deviation of the prediction performance over the permutation test (*T *= 100))

Classifier	Feature Selection	**52-20-split Validation** (50 trials)	**12-fold CV** (10 trials)	**LOOCV**	
				Accuracy	Permutation Test Results
SVM	SVMRFE	91.1	94.2	95.8	97.7 ± 1.8

SVM	L1SVM	84.8	92.1	93.1	81.5 ± 7.1

SVM_NL	SVMRFE_NL	88.7	94.3	97.2	92.4 ± 3.5

SVM_NL	SVMRW	79.4	86.8	91.7	78.3 ± 6.8

**Table 4:** Statistics on the Number of Important Features from Models Described in Table 3 (The last row lists the mean and standard deviation of the size of the feature selection results, i.e. the number of the selected features, over the permutation test (*T *= 100))

# Features	**SVMRFE**	**L1SVM**	**SVMRFE_NL**	**SVMRW**
	33	42	45	41

Permutation Test Results	39 ± 10	52 ± 4	42 ± 8	32 ± 11

The aforementioned experiments can be regarded as measuring the SVM predictive performance of certain feature subsets, regardless of how the subsets were obtained. However, Furlanello et al, 2003 [56] indicated that applying feature selection over the whole dataset might introduce selection bias into the evaluation of the feature selection results even if the prediction performance is obtained through cross-validation. Therefore, a third set of experiments to compare the generalization performance of the feature selection methods themselves in combination with SVM was performed under more conservative settings as illustrated in Figure 2(b). For each feature selection method, at each evaluation, the method was first applied only to the training dataset and then the prediction performance of the selected feature subset on the validation (test) dataset was measured. As shown in Table 5, the best prediction performance in this setting was 80.6%, which is comparable to the prediction performance without feature selection, while the feature size is reduced, on average, from 592 to 38 (with SVMRFE_NL, Table 6). LOOCV evaluation leads to a higher test accuracy than the other two evaluation procedures demonstrating the effect of the training set size on the test accuracy. LOOCV evaluation results indicate that feature selection using SVMRFE_NL achieved the best prediction performance, L1SVM was the second best feature selection method, while SVMRFE was the worst. Both 52-20-split validation and 12-fold CV evaluation results indicate that L1SVM performed the best, SVMRFE_NL performed the second best, and SVMRW resulted in the worst prediction accuracy. Overall, a clear winner was not easily identifiable among the tested methods.

**Table 5:** Prediction Performance (%): Feature Selection Methods Applied to Training Subsampling of Dataset during Each Validation (The last column lists the mean and standard deviation of the prediction performance over the permutation test (*T *= 100))

Classifier	Feature Selection	**52-20-split Validation** (50 trials)	**12-fold CV** (10 trials)	**LOOCV**			
				Accuracy	Sensitivity	Specificity	Permutation Test Results
SVM	SVMRFE	67.7	71.4	69.4	70.3	68.6	-

SVM	L1SVM	72.9	76.8	76.4	78.4	74.3	49.8 ± 7.4

SVM_NL	SVMRFE_NL	71.6	74	80.6	83.8	77.1	-

SVM_NL	SVMRW	61.9	68.2	70.8	67.6	74.3	-

**Table 6:** Statistics on the Average Number of Important Features of the Models Described in Table 5

Classifier	Feature Selection	**52-20-split Validation** (50 trials)	**12-fold CV** (10 trials)	**LOOCV**
SVM	SVMRFE	22 ± 9	27 ± 9	28 ± 7

SVM	L1SVM	34 ± 2	41 ± 2	43 ± 1

SVM_NL	SVMRFE_NL	26 ± 8	31 ± 8	38 ± 9

SVM_NL	SVMRW	29 ± 9	36 ± 8	40 ± 5

Experiments designed to test the effect of the bagging strategy on the prediction performance were also performed (bootstrap sampling was repeated 101 times, i.e. *T *= 101). The LOOCV evaluation results (Table S-6) indicate that bagging did not boost the best prediction performance (80.6%). Although it did improve the classification accuracy for the data with certain feature selection methods, it also reduced the classification accuracy for other cases. Due to these observations and its high computational cost, the bagging process was not evaluated in further tests.

#### Statistical Significance of Prediction and Feature Selection

The statistical confidence of the prediction performance of SVM classifiers on the multimode dataset with LOOCV evaluation was investigated using a permutation test. The statistic of interest was the observed difference in classification accuracy. Permutation test results (T = 1000) showed that the classification accuracy differences between linear SVM and a random classifier, as well as that between a polynomial kernel SVM (degree 2) and a random classifier, were statistically significant (p-value = 0), while the difference between linear SVM and polynomial kernel SVM was not (p-value = 0.32).

The statistical significance of the observed classification accuracy (Table 2, column 4) was also evaluated. This is captured by the null hypothesis (*H*_0_) where the performance statistics of a classifier on the true data are consistent with its performance statistics on the data with randomly assigned labels. The statistic of interest is the classification performance. The permutation test (T = 1000, results summarized in Table 2, column 7) showed that the results with SVM classifiers are statistically significant (p-value = 0).

**Figure 3:**
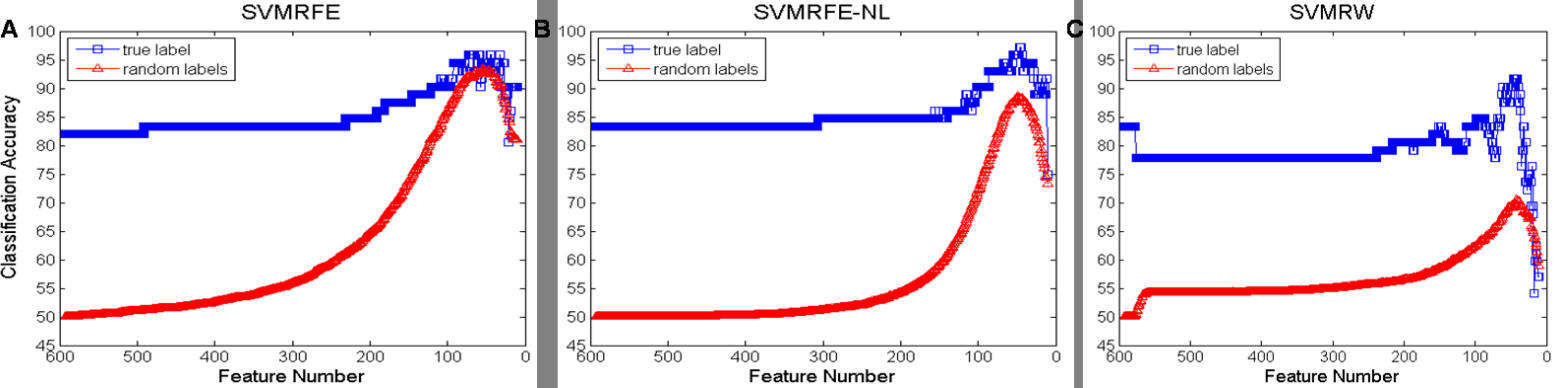
**Trends of Prediction Performance over Multimode Dataset and Random Datasets Generated over Permutation Test during Iterative Feature Selection Methods**. Comparison between the trend of the prediction performance of the multimode dataset (in blue square) and that of the averaged prediction performance of the datasets with random label permutations (in red square) to the number of remaining features during iterative feature selection methods (A) SVMRFE, (B) SVMRFE_NL, and (C) SVMRW. The x-axis is the number of remaining features, and the y-axis is prediction performance (measured by the LOOCV average accuracy).

Further assessment of the statistical significance of prediction performance subsequent to feature selection (with feature selection applied on the whole dataset, Table 3, column 5) was performed. The permutation test was designed as follows: at the *t*^*th *^test, i) a dataset *D*_*t *_was generated by random label permutation on the original dataset *D*_0_, ii) each feature selection method *A *was applied to the dataset *D*_*t *_to select an optimal feature subset *F*_*A*, *t*_, and iii) the prediction performance  on the dataset *D*_*t *_with features in *F*_*A*, *t *_was measured using LOOCV evaluation. The comparison plot of the trend of the prediction performance to the number of remaining features during iterative feature selection method (Figure 3) showed that, i) the averaged prediction performance of the datasets generated over the permutation test (*T *= 100, starts with around 50% accuracy) gradually catches up with the prediction performance of the multimode dataset (starts with over 80% accuracy) as the number of remaining features decreases; ii) for SVMRFE, the maximal value of the averaged prediction performance of the datasets generated over the permutation test (93.4% ± 4.5% when the feature size decreases to 55 on average) is close to the best prediction performance of the multimode dataset (95.8% when feature size decreases to 33); iii) for SVMRFE_NL, the best average prediction performance of the datasets with random label permutations (88.5% ± 4.9%) is comparable to the best prediction performance of the multimode dataset (97.2%); iv) for SVMRW, the best prediction performance of the multimode dataset (91.7%) is much better than that of the random datasets (70.5% ± 9.0%). As quantified in Table 3, column 6, the permutation results indicate a p-value of 0.94 for SVMRFE (i.e. for 94% of the dataset with random label permutation, the method was able to find a feature subset that achieves at least as good a classification accuracy as it did on the original dataset); while SVMRFE_NL had a p-value of 0.11. These results demonstrated the effect of selection bias in feature selection as indicated by Furlanello et al, 2003 [56]. Therefore, these feature selection methods were further evaluated through validation. L1SVM (p-value = 0.04) and SVMRW (p-value = 0.02) appeared to be less affected by selection bias.

A statistical comparison between the tested feature selection methods was performed to determine if SVMRFE_NL > SVMRFE > L1SVM > SVMRW, as observed in previous experiments. (*A *> *B *denotes that the feature selection results of method *A *generally outperform that of method *B *in prediction accuracy.) The descriptor used in this permutation test was , the difference between the prediction performance on the dataset with the feature subset output by methods *A *and *B*, respectively. The prediction performance difference between the SVMRFE_NL and SVMRFE methods was statistically significant (p-value = 0.01, *T *= 100) while the other observed prediction performance differences were not. These results were probably affected by selection bias due performing feature selection on the whole dataset, therefore, a statistical comparison between feature selection methods was also conducted in a more conservative way, i.e. through validation, as described below.

**Figure 4:**
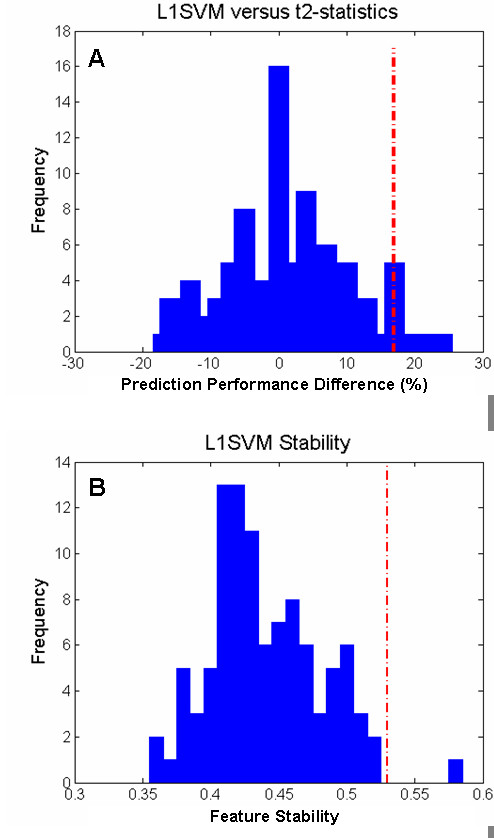
**Empirical Distribution over Permutation Test on Performance of Feature Selection Method**. Plots showing the (A) prediction performance difference between the feature selection results of L1SVM and that of t2-statistics (same number of selected features), and (B) stability of L1SVM. The x-axis is the statistic of interest of the corresponding permutation test. The y-axis is the frequency of the given value of the statistic of interest. The red dotted line indicates the observed statistic of interest and a blue bar describes the frequency at a given value of the statistic of interest from the permutation test.

The statistical significance of prediction performance subsequent to feature selection in the more conservative setting (with feature selection applied only to the training subsampling of each cross-validation, Table 5, column 5) was also assessed. First, the feature selection methods were applied to the training subsampling of the dataset to determine the optimal feature subset. Next, the prediction accuracy on the test subsampling of the dataset (nonoverlapping with the training subsampling) was obtained using the SVM model built on the training subsampling with only the selected features. The statistic of interest is the average prediction accuracy over the LOOCV procedure. The permutation test (*T *= 100) showed that the feature selection results of L1SVM were statistically significant (p-value = 0, see Table 5, column 8). Due to the heavy workload of the involved computations for the iterative methods SVMRFE, SVMRFE_NL and SVMRW over LOOCV evaluation, permutation tests to analyze the statistical significance of these methods were not conducted. Instead, L1SVM was compared with t2-statistics. In this statistical comparison, for each validation of LOOCV evaluation process, L1SVM was applied to the training set to select out *k *features and the prediction accuracy on the test set with these *k *features was obtained. Next, another set of *k *features using t2-statistics computed on the training set was selected and the prediction accuracy of the test set with the selected features was measured. The results (*T *= 100) showed that the prediction performance differences between the feature selection results of L1SVM (76.4%) and t2 statistics (59.7%) could be considered statistically significant (p-value = 0.08, empirical distribution of the statistic of interest is described in Figure 4(a)).

**Figure 5:**
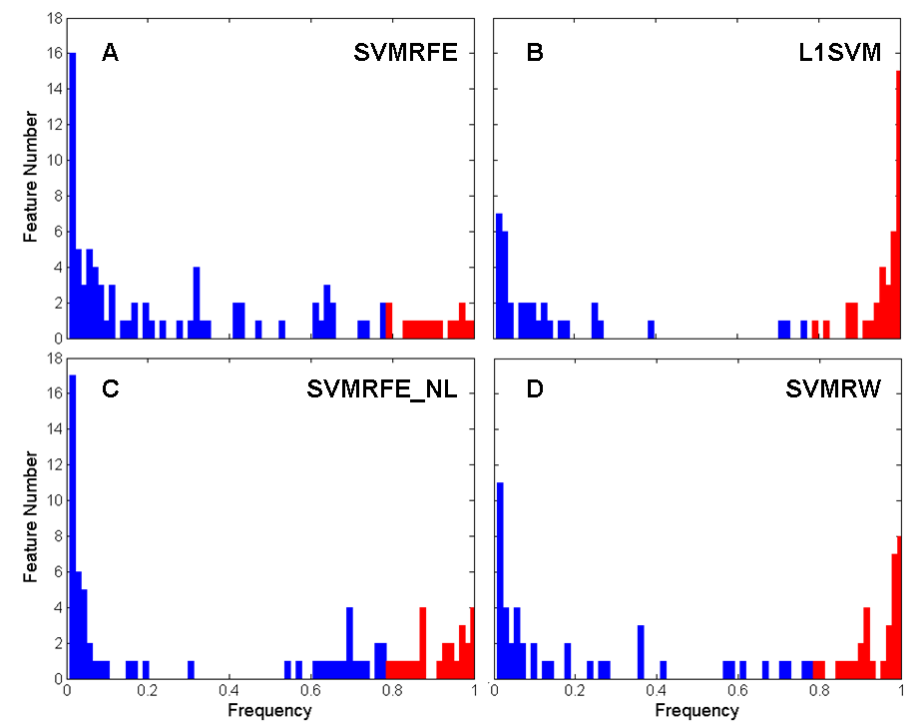
**Distribution of Feature Occurrence Frequency over the LOOCV Evaluation**. Distribution of the frequency of the features that occur in the feature selection results over the LOOCV evaluation for (A) SVMRFE, (B) SVMRFE_NL, (C) L1SVM, and (D) SVMRW methods. The bar indicates the number of features at a given feature occurrence frequency (red when frequency = 0.8 otherwise blue).

For completeness, the stability of the feature selection results over the LOOCV folds was evaluated. At each cross-validation, a feature subset was obtained; hence the frequency of occurrence of features in these feature subsets was collected. Utilizing this frequency required the concepts of *stable features*, features with an occurrence frequency over a certain threshold (80% was used here), and *stability*, the ratio of stable features in the union of the selected feature subsets during cross-validations. The distribution of feature occurrence frequency over the LOOCV feature selection results are described in Figure 5, out of the 73 features selected by L1SVM during LOOCV evaluation, 39 were found to be stable (53.4% stability), SVMRFE had 16 stable features out of 90 (stability of 17.8%), SVMRFE_NL had 26 stable features out of 82 (stability of 31.7%) and SVMRW had 33 stable features out of 77 (stability 42.9%). The prediction performance of these stable features (measured by LOOCV averaged accuracy) was 93.1% for L1SVM, 84.7% for SVMRFE_NL, 83.3% for SVMRFE and 81.9% for SVMRW. The statistical significance of the features' stability [57] was further evaluated using the stability statistics of feature selection results on the data with random label permutation over the LOOCV evaluation process as the statistic of interest. The results of the permutation tests (T = 100) show that the stability of the L1SVM method was statistically significant with a p-value of 0.01 (empirical distribution see Figure 4(b)). Because of the intensive computations involved, statistical analyses of stability for the SVMRFE, SVMRFE_NL and SVMRW methods were not performed.

### Metabolite Identification on Selected Features

The calculated neutral masses, species investigated, and retention times of the positive and negative ion mode ESI variables used by the multimode SVMRFE_NL and L1SVM models are reported in Table S-7 [see Additional file 1]. These models consist of the stable features (threshold 80%) obtained over the LOOCV evaulation as described above. Adduct analysis of the 26 stable SVMRFE_NL features provided 19 unique features to search against the databases while a similar analysis of the 39 stable L1SVM features provided 25 unique features. Comparison of the stable, unique SVMRFE_NL and L1SVM features revealed a total of 19 overlapping features. As identification of mass spectral features obtained using single-stage mass analyses is extremely difficult due to the presence of isobaric species, only tentative identifications based on comparison of the accurate mass and isotopic ratios of the selected features are provided. Therefore, validation of the chemical formulae, mass differences (Δm), matching scores, and identifications listed in Table S-7 for each of the stable, unique features is outside the scope of these experiments.

Twelve of the SVMRFE_NL-selected features from the multimode dataset were tentatively identified as endogeneous carboxylic acids, peptides, glycerophospholipids and hormones. The chemical formulae corresponding to these twelve features yielded a total of 168 possible compounds with the total number of isomers attributed to each feature ranging from 1–32, mass accuracies between 0.1–15.0 ppm and matching scores between 42.6–99.3%. Two of the identified features could not be assigned to a single chemical formulae due to the absence of additional supporting adduct ions in their respective mass spectra. One of these features was attributed to either lithocholic acid glycine conjugate or any of eight glycerophosphocholine isomers while the other was attributed to either any of eighteen glycerophosphocholine lipids containing a single double bond or to any of thirty-two lipids containing four double bonds. Examples of some of the other compounds that could be tentatively matched to the elemental formulae obtained in this investigation include 12-hydroxy-8E,10E-heptadecadienoic acid, palmitic acid, stearic acid, GlnHisAla, DHEA sulfate, PC(P-16:0/0:0), PC(10:0/4:0), PE(9:0/10:0), LysoPC(18:2(9Z,12Z)), PE-NMe(18:1(19E)/18:1(9E)). PC(14:0/20:1(11Z)), PC(14:0/22:4 (7Z,10Z,13Z,16Z)), and PC(14:0/22:1(13Z)).

Of the thirteen L1SVM-selected features that could be tentatively identified, twelve were also selected by the SVMRFE_NL model. The final unique feature, which had an accuracy of 14.8 ppm and a matching score of 98.8, was attributed to any of eleven bile acid isomers, such as 5*β*-chol-9(11)-en-24-oic acid. Although metabolites such as lysophosphatidic acid and lipid associated sialic acid, that have been investigated as metabolic biomarkers for ovarian cancer in literature [7-14], were not pinpointed in the study, the presence of several endogenous lipids as well as other endogenous metabolites in the set of selected features suggests that this approach has merit and should be further explored. Confirmation of the annotation of the metabolites and identification of remaining features selected by the SVMRFE_NL and L1SVM models will require additional accurate mass MS/MS and ^1^H-NMR experiments, and exceeds the scope of this study.

## Conclusion

The results presented here demonstrate for the first time that LC/TOF MS-based serum metabolomic experiments, in combination with state-of-the-art machine learning methods, have the potential to generate metabolic fingerprints of ovarian cancer with diagnostic applications. LOOCV generally led to a higher test accuracy than the 12-fold CV evaluation and 52-20 split validation processes, illustrating the effect of training set size on the test accuracy under this low sample number setting. Under LOOCV, classification of this serum sample test set over the selected set of features was over 90% accurate and the feature selection result of SVMRFE_NL had the best prediction accuracy (97.2%). Furthermore, prediction results obtained under the conservative settings indicated that feature selection results of SVMRFE_NL method had the best generalization performance (80.6% with feature size reduced from 592 to 38 on average). It is worth noting that L1SVM method led to good generalization performance under all three evaluation processes.

The statistical confidence of the prediction performance results by these methods was evaluated and the general performance of the feature selection methods was extensively tested. The statistical tests showed that prediction performance of the SVM/SVM_NL classifiers are significantly better than a random classifier, however, the observed prediction performance difference between the SVM_NL classifier and the SVM classifier is not statistically significant. The statistical tests on feature selection methods showed that selection bias could be introduced if feature selection methods are applied to the whole dataset (especially for SVMRFE/SVMRFE_NL methods). This might affect the prediction performance comparison between feature selection methods under this setting, because, according to the statistical tests, the observed prediction performance between any ordered pair of the four feature selection methods are not statistically significant except for that between the SVMRFE_NL and the SVMRFE method. If the feature selection methods are evaluated under the conservative settings (with method applied on the training subsampling, and feature selection results evaluated on the test/validation subsampling), the statistical test results showed that i) the prediction performance of L1SVM feature selection results was statistically significant, ii) the observed prediction performance difference between L1SVM and t2-statistics was statistically significant, and iii) the observed stability of the feature selection results of L1SVM was statistically significant. Due to the expensive computational costs of SVMRFE, SVMRFE_NL and SVMRW methods, statistical analyses of their generalization performance were not conducted.

Future studies with larger sample sets will allow the testing of more sophisticated machine learning methods with various object classes, including objects grouped by cancer stages. In addition, utilizing electrospray ion sources with rapid switching polarity and ultrahigh pressure (UPLC) separations would optimize throughput and increase the utility of our approach for diagnostic purposes. The use of higher mass accuracy and resolving power instruments, coupled to accurate mass MS/MS experiments to identify all metabolites in diagnostic panels and aid in distinguishing between isomers, are additional future directions that necessitate more advanced machine learning methods.

## Authors' contributions

FMF, AG, and JFM conceived the study. JFM, LDW and BBB provided the samples and patient information. FMF provided expertise on mass spectrometric analyses. AG provided expertise on machine learning and validation methods. WG carried out all computations and performed statistical testing. MZ developed the sample pretreatment and LC/TOF MS protocols, and conducted all sample analysis. CYH provided assistance with data processing and metabolite identification. All authors equally contributed to the drafting of the article, and read and approved the final manuscript.

## Supplementary Material

Additional file 1 **Detailed experimental protocols, supplementary analysis of results, and metabolite identification**. The data provide Method S-1, a detailed description of the sample pretreatment and LC/TOF MS analysis protocols including the LC program used (Table S-1); Analysis S-1, the prediction performance results for the pos-ion-mode and neg-ion-mode datasets (Table S-2 through S-5); Analysis S-2, results for the effect of the bagging strategy on the prediction performance over LOOCV evaluation (Table S-6); Table S-7, a list of metabolites tentatively identified from the set of stable (80%) threshold, unique SVMRFE_NL and L1SVM features, and Figure S-1, the corresponding mass spectra. Click here for file

Additional file 2 **Dataset files**. This compressed folder consists of Dataset.mat (a Matlab file containing all datasets and labels), multimode.txt, pos-ion-mode.txt, neg-ion-mode.txt, row label.txt, and column label.txt. The row label text file lists the classes used (N = Controls, the Roman numeral indicates the cancer stage, and R = recurring). The column label text file lists the complete set of features ordered into four columns: i) feature index, ii) ionization mode, iii) retention time (*min*), and iv) *m/z*. Click here for file
