# Supplementary material for: Ovarian cancer detection from metabolomic liquid chromatography/mass spectrometry data by support vector machines
Source: BMC Bioinformatics. 2009 Aug 22;10:259. doi: 10.1186/1471-2105-10-259 (PMC2741455; doi:10.1186/1471-2105-10-259)
Supplement: Additional file 1 — Detailed experimental protocols, supplementary analysis of results, and metabolite identification. The data provide Method S-1, a detailed description of the sample pretreatment and LC/TOF MS analysis protocols including the LC program used (Table S-1); Analysis S-1, the prediction performance results for the pos-ion-mode and neg-ion-mode datasets (Table S-2 through S-5); Analysis S-2, results for the effect of the bagging strategy on the prediction performance over LOOCV evaluation (Table S-6); Table S-7, a list of metabolites tentatively identified from the set of stable (80%) threshold, unique SVMRFE_NL and L1SVM features, and Figure S-1, the corresponding mass spectra. [file 1471-2105-10-259-S1.doc]

**Additional file 1: Detailed experimental protocols, supplementary analysis of results, and metabolic Identification**

**Method S-1**: Detailed Description of Sample Pretreatment and LC/TOF MS Protocols

Serum samples were thawed, and proteins precipitated by addition of acetonitrile to the serum sample in a 5:1 ratio (1000 μL acetonitrile + 200 μL serum). The mixture was vortexed for 1 minute and incubated at room temperature for 40 minutes, then the sample was centrifuged at 13,000 g for 15 minutes and the supernatant retained. The supernatant was vacuum evaporated and the residue reconstituted in 80% acetonitrile/0.1% TFA.

LC/TOF MS analyses were performed on a JEOL AccuTOF (Tokyo, Japan) mass spectrometer coupled to an Agilent 1100 Series LC system (Santa Clara, CA) via an ESI source. The TOF resolving power measured at full width half maximum was 6000 and the observed mass accuracies ranged from 5-15 ppm, depending on the signal-to-noise ratio of the particular ion investigated. The LC system was equipped with a solvent degasser, a binary pump, an autosampler, and a thermostatic column compartment (held at 25ºC). The injection volume was 15 μL in all cases. Reverse phase separation of serum samples was performed using a Symmetry® C18 column (3.5 μm, 2.1 x 150 mm, pore size 100Å; Waters, Milford, MA) at a flow rate of 150 μL min^-1^. The analytical column was preceded by a Zorbax® RX-C18 guard column (5.0 μm, 4.6 x 12.5 mm, pore size 2 μm; Agilent). The LC solvent mixtures used were: A = 0.1% formic acid in water and B = 0.1% formic acid in acetonitrile. After a pre-run wash and equilibration with 5% B for 15 minutes, data acquisition was started and the solvent composition was varied according to the solvent program described in the Table S-1. After analysis of a given serum specimen, a 0.20 mM sodium trifluoroacetate standard (NaTFA) was run for mass drift compensation purposes. For NaTFA analysis, 100% B at a flow rate of 300 μL min^-1^ was used and data was acquired for 10 minutes. After each injection of the sample or drift correction standard, the column was washed with 100% B for 30 minutes.

**Table S-1:** LC solvent gradient used in metabolomic experiments.

| Time (min) | %B  (acetonitrile/0.1% formic acid) | Flow Rate (μLmin^-1^) |
| --- | --- | --- |
| Pre-Run Column Equilibration | | |
| 0.0 | 100 | 300 |
| 10.0 | 5 | 150 |
| 15.0 | 5 | 150 |
| Sample Run | | |
| 0.0 | 5 | 150 |
| 5.0 | 5 | 150 |
| 10.0 | 20 | 150 |
| 20.0 | 25 | 150 |
| 28.0 | 30 | 150 |
| 38.0 | 35 | 150 |
| 50.0 | 40 | 150 |
| 90.0 | 45 | 150 |
| 100.0 | 50 | 150 |
| 110.0 | 60 | 150 |
| 120.0 | 75 | 150 |
| 130.0 | 85 | 150 |
| 160.0 | 95 | 150 |
| 180.0 | 100 | 150 |
| Post-Run Column Wash | | |
| 0.0 | 100 | 300 |
| 30.0 | 100 | 300 |
| NaTFA Standard Run | | |
| 0.0 | 100 | 300 |
| 10.0 | 100 | 300 |
| Post-Run Column Wash | | |
| 0.0 | 100 | 300 |
| 30.0 | 100 | 300 |

Spectral data was collected in the 100-1750 *m/z* range with a spectral recording interval of 1.5 s and a data sampling interval of 0.5 ns for both positive and negative ion ESI modes. The settings for the TOF mass spectrometer for positive or negative ion mode were as follows: needle voltage: +/- 2000 V, ring lens: +8 V or -9 V, orifice 1: +30 V or -69 V, orifice 2: +6 V or -8 V, desolvation chamber temperature: 250ºC, orifice 1 temperature: 80ºC, nebulizing gas flow rate: 1.0 L min^-1^, desolvation gas flow rate 2.5 L min^-1^, and detector voltage +/- 2800 V. The TOF analyzer pressure was 4.8 E-6 Pa during analysis. The RF ion guide voltage amplitude was swept to ensure adequate transmission of analytes in a wide range of *m/z* values. The sweep parameters were as follows: initial peaks voltage: 700 V, initial time: 20%, sweep time: 50%, final peaks voltage: 2500 V. After LC/TOF MS data was collected, it was centroided, mass drift corrected using the NaTFA reference spectrum, and exported in NetCDF format for further mining.

To ensure maximum reproducibility in metabolomic experiments, all serum specimens were run consecutively within a 2.5 month period. Every cancer sample was randomly paired with a normal sample and run on the same day to ensure that no temporal bias was introduced in the way samples were analyzed. Sample pairs were run in random order and in duplicate.

**Analysis S-1:** Detailed Description of the Prediction and Feature Selection Performance for the Pos-Ion-Mode and Neg-Ion-Mode Datasets

The prediction performance of the pos-ion-mode and neg-ion-mode datasets evaluated without feature selection are summarized in Table S-2. As apparent in the table, the neg-ion-mode dataset had a better prediction performance than the pos-ion-mode dataset, with the highest prediction performance (81.9%) obtained using the linear SVM classifier. For the pos-ion-mode dataset, the nonlinear SVM classifier generally outperformed the linear SVM classifier, while for the neg-ion-mode dataset, the linear SVM generally had a better performance.

**Table S-2: Prediction Performance (%) without Feature Selection**

| **Classifier** | **52-20-split Validation** | **12-fold CV** | **LOOCV** | | |
| --- | --- | --- | --- | --- | --- |
|  | (50 trials) | (10 trials) | Accuracy | Sensitivity | Specificity |
| *pos-ion-mode (n = 360)* | | | | | |
| **SVM** | 70 | 71.3 | 72.2 | 64.9 | 80 |
| **SVM_NL** | 71.8 | 75.6 | 73.6 | 78.4 | 68.6 |
| *neg-ion-mode (n = 232)* | | | | | |
| **SVM** | 73.2 | 80.4 | 81.9 | 81.1 | 82.9 |
| **SVM_NL** | 72.4 | 79.9 | 80.6 | 81.1 | 80 |

Table S-3 summarizes the prediction performance of the pos-ion-mode and neg-ion-mode datasets subsequent to feature selection (Figure 2a, where each feature selection method was applied to the whole dataset, then the prediction performance of the dataset containing only the selected feature subset (panel) was measured using the three evaluation processes) and gives the number of important features identified for each model. The estimated predictive performance was surprisingly high (greater than 90% for most of the methods) under LOOCV, which is perhaps the most accurate evaluation technique in this low-sample setting. For the pos-ion-mode and neg-ion-mode datasets, the feature selection results of SVMRFE had the best discriminative power followed by that of SVMRFE_NL method, while SVMRW performed the worst.

**Table S-3:** Prediction Performance (%) and the Number of Important Features for the SVM and SVM_NL Classifiers Evaluated After Feature Selection is Applied to the Whole Dataset

| **Classifier** | **Feature Selection** | **52-20-split Validation** | **12-fold CV** | **LOOCV** | **# Important Features** |
| --- | --- | --- | --- | --- | --- |
|  |  | (50 trials) | (10 trials) |  |  |
| *pos-ion-mode (n = 360)* | | | | | |
| **SVM** | **SVMRFE** | 81.6 | 87.6 | 91.7 | 36 |
| **SVM** | **L1SVM** | 72.9 | 75.1 | 76.4 | 36 |
| **SVM_NL** | **SVMRFE_NL** | 76.2 | 81.1 | 83.3 | 22 |
| **SVM_NL** | **SVMRW** | 60.5 | 61.3 | 65.3 | 32 |
| *neg-ion-mode (n = 232)* | | | | | |
| **SVM** | **SVMRFE** | 94.0 | 98.5 | 100.0 | 47 |
| **SVM** | **L1SVM** | 82.5 | 91.8 | 95.8 | 46 |
| **SVM_NL** | **SVMRFE_NL** | 88.5 | 95.7 | 97.2 | 23 |
| **SVM_NL** | **SVMRW** | 77.4 | 83.3 | 88.9 | 32 |

Table S-4 shows a comparison of the prediction performance of SVM in combination with feature selection methods performed under more conservative settings (Figure 2b, where at each evaluation, the feature selection method was first applied to a training dataset and then the prediction performance of the selected feature subset on the test dataset was measured) while Table S-5 shows the average number of important features identified in these models. The best prediction performance of the pos-ion-mode and neg-ion-mode datasets in this setting is 80.6%, which is comparable to the prediction performance without feature selection. The feature size is reduced from 232 to 41 (with SVMRFE on neg-ion-mode dataset using LOOCV). LOOCV evaluation leads to a higher test accuracy than the other two evaluation procedures demonstrating the effect of the training set size on the test accuracy. LOOCV evaluation results indicate that feature selection using SVMRFE achieved the best prediction performance, the L1SVM method was the second best feature selection method while SVMRW was the worst. Both 52-20-split validation and 12-fold CV evaluation results indicate that i) L1SVM performed the best on the neg-ion-mode datasets, ii) SVMRFE_NL method performed the best on the pos-ion-mode dataset, and iii) SVMRW method resulted in the worst prediction accuracy. Overall, a clear winner was not easily identifiable among the tested methods.

**Table S-4:** Prediction Performance (%) Evaluated After Feature Selection is Applied to Training Subsampling of Dataset during Each Validation

| **Classifier** | **Feature Selection** | **52-20-split Validation** | **12-fold CV** | **LOOCV** | | |
| --- | --- | --- | --- | --- | --- | --- |
|  |  | (50 trials) | (10 trials) | Accuracy | Sensitivity | Specificity |
| *pos-ion-mode (n = 360)* | | | | | | |
| **SVM** | **SVMRFE** | 64.0 | 67.5 | 72.2 | 64.9 | 80.0 |
| **SVM** | **L1SVM** | 65.5 | 70.6 | 70.8 | 70.3 | 71.4 |
| **SVM_NL** | **SVMRFE_NL** | 66.5 | 71.4 | 66.7 | 73.0 | 60.0 |
| **SVM_NL** | **SVMRW** | 60.2 | 59.7 | 59.7 | 62.2 | 57.1 |
| *neg-ion-mode (n = 232)* | | | | | | |
| **SVM** | **SVMRFE** | 68.4 | 74.7 | 80.6 | 86.5 | 74.3 |
| **SVM** | **L1SVM** | 71.5 | 76.2 | 75.0 | 83.8 | 65.7 |
| **SVM_NL** | **SVMRFE_NL** | 69.1 | 74.3 | 73.6 | 78.4 | 68.6 |
| **SVM_NL** | **SVMRW** | 59.6 | 63.6 | 69.4 | 70.3 | 68.6 |

**Table S-5:** Statistics on the Average Number of Important Features of the Models Described in Table S-4

| **Classifier** | **Feature Selection** | **52-20-split Validation** | **12-fold CV** | **LOOCV** |
| --- | --- | --- | --- | --- |
|  |  | (50 trials) | (10 trials) |  |
| *pos-ion-mode (n = 360)* | | | | |
| **SVM** | **SVMRFE** | 25 ± 7 | 31 ± 8 | 35 ± 5 |
| **SVM** | **L1SVM** | 30 ± 2 | 35 ± 2 | 36 ± 1 |
| **SVM_NL** | **SVMRFE_NL** | 21 ± 7 | 30 ± 10 | 26 ± 7 |
| **SVM_NL** | **SVMRW** | 20 ± 9 | 27 ± 11 | 31 ± 9 |
| *neg-ion-mode (n = 232)* | | | | |
| **SVM** | **SVMRFE** | 27 ± 9 | 33 ± 8 | 41 ± 9 |
| **SVM** | **L1SVM** | 34 ± 2 | 41 ± 2 | 44 ± 2 |
| **SVM_NL** | **SVMRFE_NL** | 33 ± 8 | 37 ± 7 | 36 ± 9 |
| **SVM_NL** | **SVMRW** | 32 ± 10 | 34 ± 7 | 34 ± 7 |

**Analysis S-2:** Detailed Description of the Effect of Bagging on Prediction Performance

The effects of the bagging strategy (bootstrap sampling was repeated 101 times, i.e. *T=101*) on the prediction performance for the multimode, pos-ion-mode, and neg-ion-mode datasets under LOOCV evaluation are summarized in Table S-6. The results indicate that bagging does not boost the best prediction performance (80.6%). Although it did improve the classification accuracy for the data with certain feature selection methods (highlighted in bold), it also reduced the classification accuracy for other cases (highlighted in italics). Due to these observations and its high computational cost, the bagging process was not evaluated in further tests.

**Table S-6:** Averaged LOOCV Prediction Performance with Bagging (%): Feature Selection Methods Applied to Training Subsampling of Dataset

| **Performance** | **SVMRFE** | **L1SVM** | **SVMRFE_NL** | **SVMRW** |
| --- | --- | --- | --- | --- |
| *multimode (n = 592)* | | | | |
|  | **72.2** | **79.2** | 80.6 | 70.8 |
| *pos-ion-mode (n = 360)* | | | | |
|  | *70.8* | **73.6** | *65.3* | **61.1** |
| *neg-ion-mode (n = 232)* | | | | |
|  | 80.6 | *70.8* | **76.4** | *66.7* |

**Table S-7:** Tentative identifications for stable (80% threshold) L1SVM and SVMRFE_NL-selected features from the multimode dataset.^A^

**Table S-7, cont’d:** Tentative identifications for stable (80% threshold) L1SVM and SVMRFE_NL-selected features from the multimode dataset.^A^

**Table S-7, cont’d:** Tentative identifications for stable (80% threshold) L1SVM and SVMRFE_NL-selected features from the multimode dataset.^A^

**Table S-7, cont’d:** Tentative identifications for stable (80% threshold) L1SVM and SVMRFE_NL-selected features from the multimode dataset.^A^

**(A)** Matches to identified compounds were made using accurate mass measurements and isotope cluster matching. For species which could not be matched against metabolite databases, the top matching formulae (according to score) are listed (for features matching fewer than five formulae, all formulae are shown). For features corresponding to multiple possible isomers, the following nomenclature is given: # isomers found including name of isomer *[source (cross-listed source, if any)].*

**(B)** Adduct analysis yielded multiple possible ion species for this feature. All are listed as none could be matched against the databases.

**(C)** 16 isomers found including **palmitic acid** *[LMFA 01010001 (HMDB 00220)],* **isopalmitic acid** *[LMFA 01020010],* **2,6-dimethyl-tetradecanoic acid** *[LMFA 01020038],* **2,8-dimethyl-tetradecanoic acid** *[LMFA 01020039],* **3-methyl-pentadecanoic acid** *[LMFA 01020164],* **2-propyl-tridecanoic acid** *[LMFA 01020165],* **2-hexyl-decanoic acid** *[LMFA 01020166],*  **3-ethyl-3-methyl-tridecanoic acid** *[LMFA 01020167],* **2-heptyl-nonanoic acid** *[LMFA 01020168],* **6-ethyl-tetradecanoic acid** *[LMFA 01020169],*  **2,4-dimethyl-tetradecanoic acid** *[LMFA 01020170],* **3,5-dimethyl-tetradecanoic acid** *[LMFA 01020171],* **4-hexyl-decanoic acid** *[LMFA 01020172],*  **2-ethyl-2-butyl-decanoic acid** *[LMFA 01020173],*  **13-methyl-pentadecanoic acid** *[LMFA 01020192],* **4,8,12-trimethyl-tridecanoic acid** *[LMFA 01020249].*

**(D)** Adduct analysis yielded multiple possible ion species for this feature. Only 1 species could be tentatively identified*.*

**(E)** Adduct analysis yielded multiple possible ion species for this feature. Only 1 species could be tentatively identified

**Table S-7, cont’d:** Tentative identifications for stable (80% threshold) L1SVM and SVMRFE_NL-selected features from the multimode dataset.^A^

**(F)** 12 isomers found including **stearic acid** *[HMDB 00827 (LMFA 01010018, MID 189, MMCD cq_00998)],* **10-methyl-heptadecanoic acid** *[MID 4292 (LMFA 01020013)],*  **(+)-isostearic acid** *[MID 4293 (LMFA 01020014)],* **2,6-dimethyl-hexadecanoic acid** *[MID 4324 (LMFA 01020042)],* **4,8-dimethyl-hexadecanoic acid** *[MID 4325 (LMFA 01020043)],* **2,14-dimethyl-hexadecanoic acid** *[MID 4326 (LMFA 01020044)],* **4,14-dimethyl-hexadecanoic acid** *[MID 4327 (LMFA 01020045)],* **6,14-dimethyl-hexadecanoic acid** *[MID 4328 (LMFA 01020046)],* **lambda isostearic acid** *[MID 4493 (LMFA 01020093)],* **neostearic acid** *[MID 4620 (LMFA 01020094)],* **11,15-dimethyl-hexadecanoic acid** *[MID 34604 (LMFA 01020175)],* **15-methyl-heptadecanoic acid** *[MID 34632 (LMFA 01020205)].*

**(G)** 6 isomers found including **Gln His Ala** *[MID 23091],* **Gln Ala His** *[MID 22217],* **Ala His Gln** *[MID 21229],* **Ala Gln His** *[MID 16023],* **His Gln Ala** *[MID 20595],* **His Ala Gln** *[MID 18707].*

**(H)** Adduct analysis yielded multiple possible ion species for this feature. Only 1 species could be tentatively identified*.*

**(I)** 11 isomers found including **5β-chol-9(11)-en-24-oic Acid** *[MID 42731 (LMST 04010142)]*, **5β-chol-11-en-24-oic Acid** *[MID 42732 (LMST 04010143)]*, **5β‑chol‑14-en-24-oic Acid** *[MID 42733 (LMST 04010144)]*, **5β-chol-2-en-24-oic Acid** *[MID 42757 (LMST 04010170)]*, **5β-chol-3-en-24-oic Acid** *[MID 42846 (LMST 04010263)]*, **Chol-4-en-24-oic Acid** *[MID 42847 (LMST 04010264)]*, **Chol-5-en-24-oic Acid** *[MID 42848 (LMST 04010265)],* **5β-chol-6-en-24-oic Acid** *[MID 042849 (LMST 04010266)]*, **5β-chol-7-en-24-oic Acid** *[MID 42850 (LMST 04010267)]*, **5β-chol-8-en-24-oic Acid** *[MID 42851 (LMST 04010268)]*, **5β‑chol‑8(14)-en-24-oic Acid** *[MID 42852 (LMST 04010269)].*

**(J)** Adduct analysis yielded multiple possible ion species for this feature. Only 1 species could be tentatively identified*.*

**(K)** 2 isomers found including **DHEA sulfate** *[HMDB 01032 (LMST 05020010)],* **testosterone sulfate** *[HMDB 02833].*

**(L)** Adduct analysis yielded multiple possible ion species for this feature. All are listed as none could be matched against the databases.

**(M)** Adduct analysis yielded multiple possible ion species for this feature. Only species that could be tentatively identified are listed.

**(N)** Cross-listed as MMCD cq-10750 and MID 5666.

**(O)** 8 isomers found including **PC(P-16:0/0:0)** *[HMDB 10407 (LMGP 01070006)],* **PC(O-16:1/0:0)** *[LMGP 01050100, 01050101, 01050102, 01050103, 01050104, 01070004, 01070005].*

**(P)** 10 isomers found including **PC(10:0/4:0)** *[LMGP 01010403],* **PC(12:0/2:0)** *[LMGP 01010443],* **PC(6:0/8:0)** *[LMGP 01011233, 01011234],* **PC(7:0/7:0)** *[LMGP 01011238, 01011239, 01011240],* **PC(8:0/6:0)** *[LMGP 01011248, 01011249],* **PC(9:0/5:0)** *[LMGP 01011269].*

**(Q)** Adduct analysis yielded multiple possible ion species for this feature. All are listed as none could be matched against the databases.

**Table S-7, cont’d:** Tentative identifications for stable (80% threshold) L1SVM and SVMRFE_NL-selected features from the multimode dataset.^A^

**(R)** 6 isomers found including **PE(9:0/10:0)[U]** *[MID 40490 (LMGP 02010091)],*  **PE(10:0/9:0)[U]** *[MID 40669 (LMGP 02010272)],* **PC(14:0/2:0)** *[LMGP 01010504],* **PC(8:0/8:0)** *[LMGP 01011251, 01011252, 01011253].*

**(S)** 3 isomers found including **glycoursodeoxycholic acid 3-sulfate** *[HMDB 02409 (MMCD cq_17361, MID 6670)],* **glycochendeoxycholic acid 7-sulfate** *[HMDB 02496 (MMCD cq_17159, MID 6692)],* **glycochendeoxycholate-3-sulfate** *[HMDB 02497 (MMCD cq_17507, MID 6702)].*

**(T)** 31 isomers found including **PE-NMe(18:1/18:1)** *[LMGP 02010331 (MMCD cq_17959), 02010333, 02010338, 02010350]*, **PC(16:0/18:2)** *[LMGP 01010585, 01010586, 01010587, 01010588, 01010589, 01010590, 01010591, 01010592, 01010593, 01010594, 01010595, 01010596]*, **PC(16:1/18:1)** *[LMGP 01010678, 01010680, 01010687, 01010688, 01010689]*,  **PC(17:1/17:1)** *[LMGP 01010726, 01010727, 01010728]*, **PC(18:0/16:2(2E,4E))** *[LMGP 01010745]*, **PC(18:1/16:1)** *[LMGP 01010886, 01010887]*, **PC(18:2/16:0)** *[LMGP 01010920, 01010926, 01010932, 01010933].*

**(U)** Adduct analysis yielded several possible ion species for the selected feature. Only species having tentative matches are listed.

**(V)** 18 isomers found including **PC(14:0/20:1(11Z))** *[HMDB 07879]*, **PC(16:0/18:1)** *[LMGP 01010005, 01010575, 01010576, 01010577, 01010578, 01010579, 01010580, 01010581, 01010582, 01010583, 01010584]*, **PC(16:1/18:0)** *[LMGP 01010679, 01010686]*, **PC(18:0/16:1(9Z))** *[LMGP 01010744]*, **PC(18:1/16:0)** *[LMGP 01010874, 01010884, 01010885]*.

**(W)** 32 isomers found including **PC(14:0/22:4(7Z,10Z,13Z,16Z))** *[HMDB 07889]*, **PC(16:0/20:4)** *[LMGP 01010007, 01010629, 01010630, 01010631]*, **PC(18:0/18:4)** *[LMGP 01010772, 01010773, 01010774, 01010775, 01010776]*, **PC(18:1/18:3)** *[LMGP 01010897, 01010898, 01010899]*, **PC(18:2/18:2)** *[LMGP 01010918, 01010919, 01010921, 01010922, 01010923, 01010924, 01010925, 01010927, 01010928, 01010929, 01010930, 01010937, 01010938, 01010939]*, **PC(18:3/18:1)** *[LMGP 01010949, 01010955]*, **PC(20:4/16:0)** *[LMGP 01011049, 01011050, 01011056].*

**(X)** Adduct analysis yielded several possible ion species for the selected feature. Only 1 species could be tentatively identified.

**(Y)** 22 isomers found including **PC(14:0/22:1(13Z))** *[HMDB 07887]*, **PC(16:0/20:1(11Z))** *[LMGP 01010618]*, **PC(18:0/18:1)** *[LMGP 01010749, 01010750, 01010751, 01010752, 01010753,01010754, 01010755, 01010756, 01010757, 01010758, 01010759, 01010760, 01010761, 01010762, 01010763]*, **PC(18:1/18:0)** *[LMGP 01010840, 01010875, 01010888, 01010889]*, **PC(20:1(11Z)/16:0)[U]** *[LMGP 01011037].*

**Figure S-1:** Mass spectra for L1SVM and SVMRFE_NL-selected features from the multimode dataset (structure and name of 1^st^ isomer listed is included as inset in spectra for those features that were tentatively matched to compounds).

**a**

**b**

**c**

**d**

**Figure S-1, cont’d:** Mass spectra for L1SVM and SVMRFE_NL-selected features from the multimode dataset (structure and name of 1^st^ isomer listed is included as inset in spectra for those features that were tentatively matched to compounds).

**e**

**f**

**g**

**h**

**Figure S-1, cont’d:** Mass spectra for L1SVM and SVMRFE_NL-selected features from the multimode dataset (structure and name of 1^st^ isomer listed is included as inset in spectra for those features that were tentatively matched to compounds).

**i**

**j**

**k**

**l**

**Figure S-1, cont’d:** Mass spectra for L1SVM and SVMRFE_NL-selected features from the multimode dataset (structure and name of 1^st^ isomer listed is included as inset in spectra for those features that were tentatively matched to compounds).

**m**

**n**

**o**

**p**

**Figure S-1, cont’d:** Mass spectra for L1SVM and SVMRFE_NL-selected features from the multimode dataset (structure and name of 1^st^ isomer listed is included as inset in spectra for those features that were matched to compounds).

**q**

**r**

**s**

**t**

**Figure S-1, cont’d:** Mass spectra for L1SVM and SVMRFE_NL-selected features from the multimode dataset (structure and name of 1^st^ isomer listed is included as inset in spectra for those features that were matched to compounds).

**u**

**v**

**w**

**x**

**Figure S-1, cont’d:** Mass spectra for L1SVM and SVMRFE_NL-selected features from the multimode dataset (structure and name of 1^st^ isomer listed is included as inset in spectra for those features that were matched to compounds).

**y**
